# The effect of laparoscopic ovarian drilling on pregnancy outcomes in polycystic ovary syndrome women with more than 2 in-vitro fertilization cycle failures: A pilot RCT

**DOI:** 10.18502/ijrm.v21i11.14653

**Published:** 2023-12-19

**Authors:** Ashraf Moini, Tayebeh Esfidani, Arezoo Arabipoor, Reihaneh Hosseini, Shima Mohiti, Sakineh Noor Mohammadi

**Affiliations:** ^1^Department of Gynecology and Obstetrics, Arash Women's Hospital, Tehran University of Medical Sciences, Tehran, Iran.; ^2^Breast Disease Research Center (BDRC), Tehran University of Medical Sciences, Tehran, Iran.; ^3^Department of Endocrinology and Female Infertility, Reproductive Biomedicine Research Center, Royan Institute for Reproductive Biomedicine, ACECR, Tehran, Iran.; ^4^Clinical Research Development Center, Mahdiyeh Educational Hospital, Shahid Beheshti University of Medical Sciences, Tehran, Iran.; ^5^Infertility Ward, Arash Women's Hospital, Tehran University of Medical Sciences, Tehran, Iran.

**Keywords:** Laparoscopy, General surgery, Polycystic ovary syndrome, Embryo implantation, Immunology, Pregnancy outcome.

## Abstract

**Background:** The effect of laparoscopic ovarian drilling (LOD) before in vitro fertilization/ intracytoplasmic sperm injection (IVF/ICSI) cycles on pregnancy outcomes is an unclear and challenging subject. **Objective:** To evaluate the impact of LOD before IVF/ICSI cycles on controlled ovarian stimulation and pregnancy outcomes in polycystic ovary syndrome (PCOS) women with a history of more than 2 IVF failures. **Materials and Methods:** In this randomized clinical trial, women with PCOS diagnosis who referred to Arash Women's hospital, Tehran, Iran for IVF/ICSI cycle from August 2015-January 2018 were evaluated. Eligible participants were allocated into 2 groups randomly (n = 17/each group). The participants in the LOD group (intervention) were treated with laparoscopic couture, and after one month, they underwent IVF/ICSI cycles using the gonadotropin-releasing hormone antagonist protocol. The control group had no intervention. The oocyte and embryo qualities, ovarian hyperstimulation syndrome rate, the rates of chemical and clinical pregnancy and early miscarriage, live birth, and pregnancy complications, were compared between groups. **Results:** Finally, 34 participants were evaluated. The controlled ovarian stimulation outcomes were similar between groups. The ovarian hyperstimulation syndrome rate in the LOD group was significantly lower than in the control group (p = 0.04). One case of spontaneous pregnancy was reported in the LOD group. No significant difference was observed between groups in clinical pregnancy, miscarriage, and live birth rates. The rates of pregnancy complications (gestational diabetes mellitus, preeclampsia, and preterm birth) were similar between groups. **Conclusion:** Performing LOD before IVF/ICSI cycles did not improve the pregnancy outcomes in PCOS women, a clinical trial with a larger sample size is needed to prove these results.

## 1. Introduction

Polycystic ovary syndrome (PCOS) is one of the most common endocrine disorders (8-13% of reproductive-aged women) (1) diagnosed with the Rotterdam consensus criteria (2).

Lifestyle change and administration of selective estrogen receptor modulators, including clomiphene citrate (CC) or aromatase inhibitor drugs (letrozole), are considered the first-step approach in treating women with PCOS (1). However, CC is not successful in ovulation induction in approximately 20% of cases (3). The second-stage treatment options for these women are gonadotropin therapy or laparoscopic ovarian surgery (4).

Women with PCOS show different manifestations during infertility treatment, both during induction ovulation and in vitro fertilization (IVF)-embryo transfer, than in women with normal ovulation (5). Women with PCOS diagnosis are more likely to respond highly to infertility treatment and produce many follicles with a risk of ovarian hyperstimulation syndrome (OHSS) and twin or multiple pregnancies (4, 6). Despite high-dose stimulation, some women with this condition may not respond well to ovarian stimulation and generate no or only a few dominant follicles ( < 3) with low serum estrogen levels. An alternative treatment for medical ovulation stimulation is the use of executive methods. It is accompanied by surgery and ovarian wedge resection (5). Laparoscopic ovarian drilling (LOD) appears useful in women with PCOS who do not respond to drug treatment and have no other reasons for infertility. This surgery method is a modified type of ovarian wedge resection (less invasive) during this process, the vascular stroma is destroyed (7). The purpose of this procedure is to increase follicular growth by reducing the amount of androgen and attenuating luteinizing hormone (LH) levels, and enhancing follicle-stimulating hormone (FSH) and sex hormone-binding globulin (4). Both ovarian cauterization methods have been used through laparoscopy and carbon dioxide or neodymium-doped yttrium aluminum garnet lasers to create multiple holes in the ovarian and sternal surfaces (5, 7).

Several studies have suggested LOD as a useful approach in the second line of treatment to stimulate ovulation in clomiphene-resistant PCOS individuals (8). In addition it increases the rate of pregnancy by stimulating temporary ovulation, reduces the amount of OHSS in the next IVF cycle as well as lowers the cancellation rate and improves the IVF cycle outcome (9). However, other studies found no improvement in pregnancy, miscarriage, and live birth rates (6, 10) and this issue is still under debate.

Because the effect of LOD before the IVF cycle on pregnancy outcome is unclear, this study was performed to determine the effect of LOD on the pregnancy rate in PCOS women undergoing IVF cycles who had a history of more than 2 IVF failures.

## 2. Materials and Methods 

This study is a randomized clinical trial conducted at Arash Women's hospital, Tehran, Iran from August 2015-January 2018. The infertile PCOS women undergoing IVF cycles with a history of > 2 IVF failures were screened to include in the study. PCOS diagnosis was based on Rotterdam criteria. Women aged between 18 and 39 yr, with a body mass index (BMI) of < 30, normal semen analysis in their spouses, no laparoscopic contraindications, and history of assisted reproductive technology (ART) failure at least twice or more were included in the study. Women suffering from previous ovarian surgery and co-existing endocrine diseases, including diabetes mellitus, estrogen-dependent tumors, thyroid disease, Cushing's syndrome, or congenital adrenal hyperplasia were excluded from the study.

A sample size of 17 subjects was calculated using the following statistical formula $\left(n=\left(Z_{a}/2+Z_{\beta }\right)2^{*}\left(p_{1}\left(1-p1\right)+p_{2}\left(1-p2\right)\right)/\left(p_{1}-p_{2}\right)2\right)$ assuming ${\text{p}}_{1}$ = 0.10 and ${\text{p}}_{2}$ = 0.50 with 95% of confidence level, and 80% power. Based on previous study, a sample size of 12 per group could be recommended as a pilot clinical trial (11).

The study's objective was explained to eligible participants, if they had written consent, they were randomly assigned to the study and control groups. The randomization method was based on a block randomization list designed by an epidemiologist colleague using the Stata software (LLC StataCorp, USA) version 13 and a block size of 6.

Participants' random allocation list was only available to the project epidemiologist. The random allocation process was concealed by providing 34 consecutive treatment cards placed in sealed envelopes. A random 10-digit code was written without sequence and frame on each envelope. This relevant code was the participant's identification number, and the project methodologist knew which code belongs to the particular treatment group. When the doctor announces that a case is eligible, the methodologist provides the doctor with an envelope design. The desired treatment was selected based on the type mentioned in the envelope. The outcome's evaluator was a third-party person who was unaware of the type of treatment performed. A statistician performed the data analysis unaware of all the study's processes.

For all women, before the treatment cycle, the basal serum levels of FSH and LH were measured. Then the history of the previous IVF cycle was collected. Since this study was conducted at the academic center, and the cost of performing anti*-*Müllerian hormone (AMH) was high, the antral follicular count was used instead of the AMH measurement. The intervention group was treated with a laparoscopic cutter (the ovarian cut is performed using a 40 W energy bipolar current at 4-6 points of the ovary) and underwent ART using a gonadotropin-releasing hormone (GnRH) antagonist after one month. This time frame between operations to initiating gonadotropin therapy was designed to allow for appropriate recovery from the endocrine changes because of LOD. The women in the control group underwent an ART cycle using GnRH antagonist protocol without prior laparoscopic catheterization. In both groups, daily injections of recombinant FSH (Merck-Serono 150 IU) were started from day 2 of the cycle. Then the GnRH-ant (Cetrotide Merck-Serono, or Orgalutran, MSD, Italy) was added 0.25 mg daily when the leading follicle diameter reached ≥ 13 mm in size. Human chorionic gonadotropin (hCG) (10,000 IU, Choriomon, IBSA, Switzerland) was administrated for the final oocyte triggering when at least 2 dominant follicles with 18 mm or greater in diameter were noticed in ultrasound monitoring (Philips Affiniti 70 machine with a C10-3v Pure-Wave endovaginal probe, UK). The ovum pick-up time was designed 34-36 hr after hCG injection, and then intra-cytoplasmic sperm injection (ICSI) was carried out following our clinical procedures. A maximum of 2 top-quality cleavage stage embryos were transferred into 2 groups.

The primary outcomes were considered as clinical and chemical pregnancy (approved observing of a gestational sac with fetal heartbeat in vaginal ultrasound at 6-7 wk of pregnancy or β-hCG test 14 days after transfer). The total number of metaphase II oocytes and embryos quality (based on the number of blastomeres and the percent of fragmentation, multinucleation, and symmetry on the third day after oocyte retrieval), the OHSS rate (3 and 10 days after ovarian puncture) as well as early miscarriage rates (spontaneous loss of a clinical pregnancy ≤ 12 wk of gestation), live birth rate and pregnancy complications including gestational diabetes, hypertension, preeclampsia, premature rupture of the amniotic sac before 37 wk of pregnancy and preterm delivery were considered as secondary outcomes.

### Ethical considerations

This study was approved by the Institutional Review Board and the Ethics Committee of Tehran University of Medical Sciences, Tehran, Iran (Code: IR.TUMS.REC.1394.542). Informed written consent was taken from each participant. The study protocol was registered in the Iranian Registry of Clinical Trials website and was updated on 2022-12-13.

### Statistical analysis

The Statistical Package for Social Sciences (SPSS Inc., Chicago, IL, USA) version 21.0 was applied for the final statistical analysis. The normal distribution was checked for the quantitative variables and all of them had a normal distribution. The comparisons of continuous variables between groups were provided by Students *t* test and presented as mean ± SD. The Fisher's exact test provided the comparisons of the categorical variables between groups. The significant statistical level was considered as p-value < 0.05.

## 3. Results

**Figure 1:**
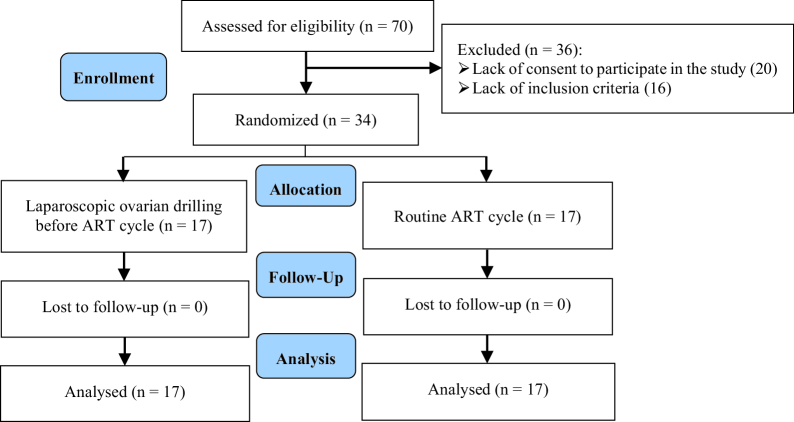
The study subjects' sampling flow chart.

Out of 70 participants screened, 20 women were not satisfied with study participation, 16 participants were not eligible, and finally, 34 participants were randomly allocated into 2 groups (Figure 1). The demographic and clinical characteristics of participants were compared between groups (Table I). No significant difference was observed between groups in terms of women's age, BMI, basal serum FSH and LH levels, antral follicular count, number of previous failed IVF cycles, and gravidity.

Table II shows the comparison of controlled ovarian hyperstimulation (COH) outcomes between groups. No significant difference was found between groups in terms of duration of stimulation, the total dose of used gonadotropins, number of used antagonist ampoules, serum estradiol on HCG day, the total number of retrieved and metaphase II oocytes, as well as total number of obtained and top-quality embryos. No cases of cycle cancellation, oocyte, or embryo development were observed in the 2 groups. The OHSS syndrome rate in the intervention group was significantly lower than the control group (6.3 vs. 35.3, p = 0.04).

The pregnancy outcomes were compared between groups in table III. One case of spontaneous pregnancy was reported in the LOD group. However, no statistically significant difference was observed between the 2 groups in clinical pregnancy, miscarriage, and live birth rates. The rates of pregnancy complications (gestational diabetes mellitus, intrauterine growth restriction, preeclampsia, preterm labor) were similar between groups.

**Table 1:** Participants' demographic and clinical characteristics


**Variables**	**LOD group (n = 17)**	**Control group (n = 17)**	**P-value**
**Age (yr) **	28.18 ± 3.98	30.70 ± 3.53	0.06
**BMI (kg/m^2^) **	25.35 ± 3.03	24.77 ± 2.38	0.54
**Duration of infertility**	4.80 ± 1.92	5.05 ± 1.86	0.15
**Gravidity **	0.56 ± 0.23	0.97 ± 0.76	0.06
**Basal FSH level (IU/L) **	7.92 ± 4.23	5.71 ± 2.30	0.07
**Basal LH level (IU/L) **	9.58 ± 4.68	8.76 ± 2.64	0.17
**No. of previous IVF**	2.47 ± 0.87	2.47 ± 0.51	0.90
Data presented as Mean ± SD. Student *t* test. LOD: Laparoscopic ovarian drilling, BMI: Body mass index, FSH: Follicle-stimulating hormone, LH: Luteinizing hormone, IVF: In-vitro fertilization			

**Table 2:** The comparison of the COH outcome between groups


**Variables**	**LOD group (n = 17)**	**Control group (n = 17)**	**P-value**
Duration of stimulation (days)*	10.43 ± 1.03	10.64 ± 1.22	0.59
No. of used antagonist ampoules*	5.68 ± 0.79	5.64 ± 0.70	0.87
Total ampoule of used gonadotropins (75IU)*	20.75 ± 2.04	21.35 ± 2.47	0.45
Serum estradiol on HCG day*	2717 ± 232.00	2738.41 ± 371.57	0.84
No. of retrieved oocytes*	14.93 ± 3.39	15.00 ± 5.52	0.96
No. of metaphase II oocytes*	11.18 ± 3.90	10.76 ± 4.64	0.78
Fertilization rate***	63.67%	65.57%	0.91
No. of obtained embryo*	7.12 ± 3.70	7.05 ± 3.94	0.96
No. of top-quality embryos*	5.1 ± 2.52	5.2 ± 2.85	0.90
OHSS rate**	1 (6.3)	6 (35.3)	0.04
All-freeze rate**	1 (6.3)	6 (35.3)	0.04
Endometrial thickness at ET day (mm)*	9.01 ± 1.28	9.08 ± 1.06	0.86
*Data presented as Mean ± SD. The Students *t* test. **Data presented as n (%). Fisher's exact test. ***Data presented as percentages. Fisher's exact test. LOD: Laparoscopic ovarian drilling, COH: Controlled ovarian hyperstimulation, HCG: Human chorionic gonadotropin, OHSS: Ovarian hyperstimulation syndrome, ET: Embryo transfer			

**Table 3:** The comparison of the pregnancy outcome between groups


**Variables**	**LOD group (n = 17)**	**Control group (n = 17)**	**P-value**
**Spontaneous pregnancy rate **	1 (5.9)	0 (0)	0.32
**Implantation rate/ET**	7 (43.8)	5 (29.4)	0.39
**Clinical pregnancy rate/ET **	6/15 (40)	4/11 (36.3)	0.38
**Miscarriage rate/ET**	1/15 (6.6)	1/11 (9.1)	0.74
**Gestational diabetes mellitus rate**	0 (0)	2 (11.8)	0.48
**Preeclampsia rate**	0 (0)	0 (0)	-
**Preterm rate**	0 (0)	0 (0)	-
**Live birth rate/ET**	6 (37.5)	4 (23.5)	0.38
Data presented as n (%). Fisher's exact test. LOD: Laparoscopic ovarian drilling, ET: Embryo transfer			

## 4. Discussion

This study showed that the use of LOD before IVF did not increase the clinical pregnancy rate; however, the OHSS and miscarriage rates were lower in this group, but the differences with the control group were not statistically significant. The mechanism of ovulation induction by the LOD, which leads to the resumption of follicular maturation and ovulation, is largely unknown (12). The most acceptable mechanism involves the thermal degradation of androgen production by Theca cells in the ovarian stroma. Decreased intravenous and peripheral androgens were found to increase FSH and decrease LH secretion and the internal flocculation environment, further promoting normal follicular maturation and ovulation. Other theories include improved ovarian blood flow with increased FSH delivery, ovarian inflammation with growth factors, and increased insulin sensitivity (13).

Several studies compared the effect of LOD with medical induction ovulation in treating PCOS women. Still, a limited clinical trial has existed regarding the impact of LOD before the ART cycle on pregnancy outcomes (3). In agreement with the present study, 50 infertile women with PCOS, who were candidates for IVF were randomly allocated into 2 groups (LOD + IVF) and (IVF only) in a clinical trial. The results showed that the pregnancy and miscarriage rates were similar between groups, but the OHSS rate in the LOD group was significantly lower than the control group (14). Elsewhere, Eftekhar et al., in a retrospective study, evaluated the treatment outcomes of 300 infertile women between 20 and 35 yr old with clomiphene-resistant PCOS who underwent IVF/ICSI cycle. The study participants were allocated into the following 2 groups based on their treatment history: group I included PCOS women who had history of LOD at least 6 months to 3 yr before IVF/ICSI (n = 150), and group II included PCOS women without history of drilling (n = 150). The antagonist protocol was used for PCOS in both groups. It was concluded that the PCOS outcomes and pregnancy rates were similar in the 2 groups. Regarding significant reduction in OHSS rate in women undergoing LOD, this surgical treatment may be considered a useful approach in managing women with a history of developed OHSS. However, some concerns regarding LOD's long-term complications on ovarian function have remained (6).

The efficacy of transvaginal ovarian drilling (TVOD) in PCOS women who had poor outcomes in more than 2 previous IVF cycles was evaluated in a prospective study. They performed TVOD before IVF for 11 cases and compared the outcomes of the current cycle with their previous IVF cycle. The results showed that the fertilization and cleavage rates in the cycle after TVOD were higher than in the previous IVF cycle. In addition, the pregnancy and the implantation rates after TVOD were same as those women with regular ovarian response who underwent IVF for tubal factor infertility during the study period. They recommended that the TVOD is effective in improving IVF outcomes in PCOS women with a history of failures in the treatment cycles, and it is less invasive and low-cost compared with LOD (15). In a pilot study, Xu et al. reported that ovulation induction from the next day after TVOD is an advantageous and convenient treatment approach for PCOS women with poor responders. The reductions of AMH and testosterone after TVOD may be the main reason for improving ovary sensitivity to gonadotropins (5). They recommended future randomized clinical studies with larger sample sizes and monitoring this strategy for long-term results. Based on the evidence that TVOD can be effective and practical, and considering that some studies have suggested the possibility of increased risk of diminished ovarian reserve or premature ovarian failure after LOD, we suggest that a clinical study be performed to compare the efficacy of these 2 procedures before IVF cycle on the COH and pregnancy outcome in PCOS women who had repeated IVF failures.

The design of the present study as a randomized clinical trial is its strength, and the results are more powerful than retrospective studies. Another strength of the study was the follow-up of pregnancies until birth. One of the weaknesses of the study is its small sample size, but due to the time and financial limitations of the study, it was not possible to study more cases. However, it is suggested to further multicenter clinical trials to evaluate the long-term effect of LOD before IVF/ICSI cycles.

## 5. Conclusion

In summary, performing LOD before the IVF cycle in PCOS women who had more than 2 IVF failures did not improve pregnancy outcomes, and only reduced the OHSS rate. According to recent studies that report on the efficiency of the TVOD method, more studies with a larger sample size should be designed to compare these 2 strategies in the treatment of infertile PCOS women before the IVF cycle.

##  Conflict of Interest

The authors declare that there is no conflict of interest.
